# Characterization of protein extracts from different types of human teeth and insight in biomineralization

**DOI:** 10.1038/s41598-019-44268-2

**Published:** 2019-06-27

**Authors:** Vaibhav Sharma, Alagiri Srinivasan, Ajoy Roychoudhury, Komal Rani, Mitali Tyagi, Kapil Dev, Fredrik Nikolajeff, Saroj Kumar

**Affiliations:** 1 Department of Biophysics, All India Institute of Medical Sciences (AIIMS), New Delhi, India; 2 Department of Biochemistry, Jamia Hamdard University, New Delhi, India; 3 Department of Oral and Maxillofacial Surgery, Center for Dental Education and Research (CDER), All India Institute of Medical Sciences (AIIMS), New Delhi, India; 4 Department of Engineering Science, Uppsala University, Uppsala, 75105 Sweden; 5 Department of Biotechnology, Jamia Milia Islamia, New Delhi, India

**Keywords:** Biophysical methods, Intrinsically disordered proteins

## Abstract

The present study describes an efficient method for isolation and purification of protein extracts from four types of human teeth i.e. molar, premolar, canine, and incisor. Detailed structural characterization of these protein extracts was done by Fourier transform infrared spectroscopy (FTIR) and circular dichroism (CD) which showed that a major fraction of the proteins present are unstructured in nature including primarily random coils in addition to the other structures like extended beta (β) structure, poly-l-proline-type II (PPII) helix, turns, with only a small fraction constituting of ordered structures like alpha (α) helix and β sheets. These resultant labile structures give the proteins the necessary flexibility that they require to interact with a variety of substrates including different ions like calcium and phosphates and for other protein-protein interactions. We also did initial studies on the mineralization of calcium phosphate with the protein extracts. Nanoparticle tracking analysis (NTA) show an increase in the size of calcium phosphate accumulation in the presence of protein extracts. We propose that protein extracts elevate the crystallization process of calcium phosphate. Our current biophysical study provides novel insights into the structural characterization of proteins from human teeth and their implications in understanding the tooth biomineralization. As per our knowledge, this is the first report which focuses on the whole protein extraction from different types of human teeth as these extracts imitate the *in vivo* tooth mineralization.

## Introduction

Biomineralization is a process which embodies the systematic accumulation of inorganic and organic materials to form hierarchical structures like bones and teeth. This process seems to be ubiquitous in nature, ranging from gold deposits in unicellular bacteria^1^ to silicates accumulation in algae and diatoms^2^, carbonates in invertebrates^3^ and finally calcium phosphates in the hard tissues of vertebrates^4^. The nature and arrangement of inorganic deposition are largely guided by the organic environment. Studies on hard tissues like teeth, shells, and bones have always been quite challenging due to numerous reasons, which include removal of excessive inorganic salts, the low yield of protein due to low abundance in such tissues, protein heterogenicity due to alternative splicing and a variety of post-translational modifications^5^.

At a molecular level, proteins are the “functional machinery” of any organism. In earlier times, it was supposed that for a protein to function its folded structure is of utmost importance, but the last 25 years have witnessed increasing reports of proteins that are unstructured yet are very much indispensable for cellular and molecular activities^6^. These proteins are termed “intrinsically disordered proteins” (IDP’s)^7^. IDPs have been reported to have a compositional bias towards the charged/polar amino acids (eg: Lys, Arg, Glu, Gln, Ser) and destitute of hydrophobic residues (eg: Tyr, Trp, Phe, Leu, Ile)^8^. These IDP’s play a crucial role in the biomineralization of tissues, including teeth and similar systems like bone and mollusk shells. IDPs are proteins whose structures are highly flexible^9^ and do not assume typical secondary structures (α-helix, β-sheet, etc.). Either these proteins have a mostly random coil-structure or may contain intrinsically disordered regions. Fourier transform infrared spectroscopy (FTIR) and circular dichroism are well-established techniques to characterize the secondary structural features of proteins^10,11^. IDPs gain more folded-features when post-translationally modified and/or when bound to their interacting partners. In the vertebrate bio-mineralization, most of the proteins associated with hydroxyapatite formation and growth are IDPs^12,13^. These IDPs may stabilize the ions or ion clusters, the critical nucleus, provide epitaxial sites for the initial mineral deposition, and/or stabilize the crystal. The flexible nature of these structures enables them to perform these and many other functions.

A tooth consist of three layer of mineralized tissues. The enamel forms the outermost layer, in which 90% organic material constitutes amelogenin, dentin forms the middle layer and cementum is the innermost layer. Tooth calcification is tightly regulated by proteins that are secreted into the extracellular matrix. The observation that unstructured proteins were involved in mineralization was first testified in 1977 in a circular dichroism (CD) study of phosphophoryn, in the presence of Ca^2+^. The addition of this ion, changed the structure from a random linear chain to a β-pleated sheet^14^. Phosphophoryn, also known as dentin phosphoprotein (DPP), is the product of the cleavage of dentine sialophosphoprotein (DSPP) into DPP and dentin sialoprotein (DSP). DSPP is the most abundant protein in dentin^15^ and is also expressed in enamel and in bone.

To date, however, most studies on *in vitro* crystallization of calcium phosphate are performed with individual proteins from enamel^16^, dentine^17^ or truncated versions of these proteins^18^. The cooperative effects of enamelin and amelogenin on crystal growth morphology was also analyzed by Janet Oldak’s group^19^. Contrary to these approaches we are reporting, for the first time, the effect of whole protein extracts from different types of teeth on *in vitro* crystallization of calcium phosphate to mimic the *in vivo* condition of tooth biomineralization.

## Materials and Methods

### Isolation of proteins from human teeth

This study was approved by the Institute Ethics Committee for Post Graduate Research of All India Institute of Medical Sciences (Ref no. IECPG-387/29.06.2016). All experiments were performed in accordance with relevant guidelines and regulations. Written informed consent was received from all participants. In the case of individuals below 18 years, informed consent was obtained from parents/legal guardian for study participation. Human teeth samples used in this study were from living, healthy individuals, which were extracted due to orthodontic reasons. The age group was 17–40 years. Samples were collected from the Center for Dental Education and Research (CDER), AIIMS, New Delhi. Collected teeth were kept at 10% NaCl to inhibit bacterial growth and contamination. Teeth were washed thoroughly with water (2–3 times). After washing, teeth were crushed in a pestle and mortar in the presence of liquid nitrogen until a fine powder was formed. 10 g of this powder was taken and mixed with 20 ml of demineralization solution containing 0.1 M EDTA, 100 mM NaCl and protease inhibitors (Roche) at 20 °C for 2 days, centrifuged at 10,000 rpm and collected supernatant was dialyzed against HEPES buffer, pH 7.4. The clarified supernatant was further concentrated by the membrane-based cut off filters (3.5 kDa), which helps to remove impurities and salts as well as also reduced the loss of smaller proteins from our extracts. The protein concentration was measured by BCA kit (Pierce) according to the manufacturer’s instructions. The samples were run on 12% SDS-PAGE and subsequently stained using silver staining.

### UV absorbance spectroscopy and circular dichroism (CD)

Absorbance measurements in the range from 200–350 nm were carried out using Cary Varian Bio 100 UV-Vis spectrophotometer (Varian Inc., California, USA). Quartz cuvettes were used for the measurements. A cell of the path length of 1.0 cm was used and a baseline correction was carried out with a blank.

CD spectra were recorded on a JASCO J-1500 spectropolarimeter (Jasco Corporation, Tokyo, Japan) using cylindrical quartz cell of path length 1 mm. The instrument was constantly purged with nitrogen. Before the experiment, the calibration of dichrometer was checked by using d-10-campher sulfonic acid. The protein concentration of 0.2 mg/ml was used. Three consecutive spectral scans were averaged and corrected by subtracting corresponding blanks. Secondary structure was monitored in the far UV region between 190 and 250 nm.

### Attenuated total reflectance Fourier transform infrared spectroscopy (ATR-FTIR)

The protein extract was placed on the SensIR ATR reflection element. Spectra were recorded by Bruker Tensor 37 equipped with a DTGS detector at 4 cm^−1^ resolution. The experiments were performed using liquid samples at room temperature. Each sample spectrum of 128 scans was ratioed against a 128 scans background spectrum. We have subtracted the buffer and water content from each sample and used the buffer as a background.Collected spectra were analyzed by OPUS software. We have also performed the water vapor correction on each spectrum. Secondary structure estimation was done by curve fitting of the amide I region by Matlab (Mathworks Inc) program Kinetics written by Erik Goormaghtigh^20^. The water vapor subtraction was done and normalized to equal band amplitudes. Before the curve fitting a straight baseline passing through the ordinate at 1700 and 1600 cm^−1^ was subtracted. The baseline was modified again by the least-squares curve-fitting program which allows for a horizontal baseline to be adjusted as an additional parameter to obtain the best fit. To determine the initial peak position for curve fitting, a second derivative spectrum was used^21^.

### Bioinformatics predictions for structural disorder

Twenty proteins were selected that were well known for their occurrence in the human tooth. Charge hydropathy (CH) plot was made. The CH plot represents a plot between mean hydrophobicity and mean net charge. The ProtParam program at the EXPASY server (https://web.expasy.org/protparam/) was used to determine the number of positively and negatively charged amino acids at pH 7 for the proteins shown on the CH-plot. The mean net charge (R) was calculated by determining the value of the absolute difference between the positively and negatively charged residues and dividing this by the total number of residues. Mean hydrophobicity was calculated by ProtScale program of EXPASY server (https://web.expasy.org/protscale/). Hydrophobicity scale of Kyte and Doolittle (1982) was chosen^22^. Plotting H versus R generated the CH-plot. The boundary line corresponds to the equation H = (R + 1.151)/2.785, which defines the boundary between disordered proteins (left side) and ordered proteins (right side)^23^.

Moreover, out of these 20 proteins, 10 were randomly selected to analyze their sequence-based disorder by two software programs, PONDR-VSL2 and IUPred^24^. These predictors have been used extensively in the literature and their performance has been thoroughly evaluated and compared to other predictors. The accuracy is on the order of 80–90%, with the software programs trained on the DisProt database. They return a score that ascribes the probability of structural disorder at residue level: a score above 0.5 in both means that the residue is likely to fall into local disorder. In the case of whole proteins, the overall disorder content was calculated by dividing the number of residues with a score higher than 0.5 by the length of the protein. A protein was considered as “mostly disordered” if more than 50% of its residues had a score above 0.5 and “fully disordered” if more than 90% of its residues fell into local disorder.

### *In vitro* mineralization studies

Stock solutions of calcium (50 mM) and phosphate (5 mM) were prepared using reagent grade CaCl_2_ (Sigma, >99.0% pure) and KH_2_PO_4_ (Sigma, >99.0% pure). All solutions (except protein extracts) were filtered (0.22-μM filters, Millipore) before use. The KH_2_PO_4_ solution was adjusted to pH 7.5–11.2 at 25 °C, using a small volume of KOH. The precise pH value was selected by design so that the reaction solution would have an initial pH ∼7.4 at 37 °C upon mixing all solution components. *A*liquots of calcium and pH-adjusted phosphate solution were sequentially added to protein solutions to yield final concentrations of 2.5 mM Ca^2+^, 1.5 mM P_i_, and 0.2–2.0 mg/mL protein, with a final volume of 100 μL, as previously reported^18^.Control experiements were also done with only protein extract (protein ageing effect) in addition to only calcium phosphate (−ve control) and with BSA. A special control for ruling out collagen biased mineralization of calcium phosphate was done with Cisplatin injured nephrotoxic rat kidney tissue sample (A kind gift from Cardiovascular Pharmacology Laboratory, AIIMS, New Delhi). ELISA was done with rat collagen 1 antibody kit (CUSA BIO) followed by a similar procedure of mineralization and NTA experiments as described above.

Samples were then placed in a thermostatic water bath adjusted to 37 °C. Initial pH values were set to ∼pH 7.4. To minimize evaporation, the reaction tube was tightly sealed with a cap or Parafilm. Each experiment was carried out using two identically prepared samples. One sample was visualized on the light microscope attached with polarization accessory and the other one proceeded for nanoparticle tracking analysis (NTA) at different time points; at 0 hours, 6 hours and 12 hours. Experiments were repeated 8–10 times.

### Enrichment of intrinsically disordered proteins in protein teeth extracts

Total soluble teeth protein extract was subjected to acid treatment by the addition of 60% Perchloric acid (PCA, Sigma) to give the desired final concentration of 3%. After incubation on ice for 15 mins, the reaction was stopped by centrifuging at 4500 g for 20 min at 4 °C^25^. After treatment and clarification, an aliquot of the soluble protein extract was kept for *in vitro* mineralization and size measurement experiment by NTA. The soluble extract was then concentrated by precipitating with 8 volumes of acetone at −20 °C overnight. Samples were run on SDS-PAGE and subsequently silver stained. The remaining pellet down fraction was also checked through *in vitro* mineralization followed by NTA size-based measurements.

### Nanoparticle tracking analysis (NTA)

NTA measurements were performed with a NanoSight LM20 (NanoSight, Amesbury, United Kingdom), equipped with a sample chamber with a 640-nm laser. The samples were injected into the sample chamber with sterile syringes (BD Discardit II, New Jersey, USA) until the liquid reached the tip of the nozzle. All measurements were performed at room temperature. The software used for capturing and analyzing the data was the NTA 2.0 Build 127. The values obtained were concentration (particles/ml) and size and these were plotted in SigmaPlot 12.0 software.

### Statistical analysis

Statistical analysis was performed with Stata 12.1 (Texas, USA). Data represent mean ± SD of 4 independent experiments. Parametric data were analyzed by two sample Student’s unpaired t-test (mean comparison test) between control vs different protein extract at all-time points. P values less than 0.05 were considered statistically significant (*<0.05; **<0.001; ***<0.0001). Graphs were plotted by using GraphPad Prism; Version 5.03; San Diego, CA 92108.

## Results and Discussion

**Figure 1:**
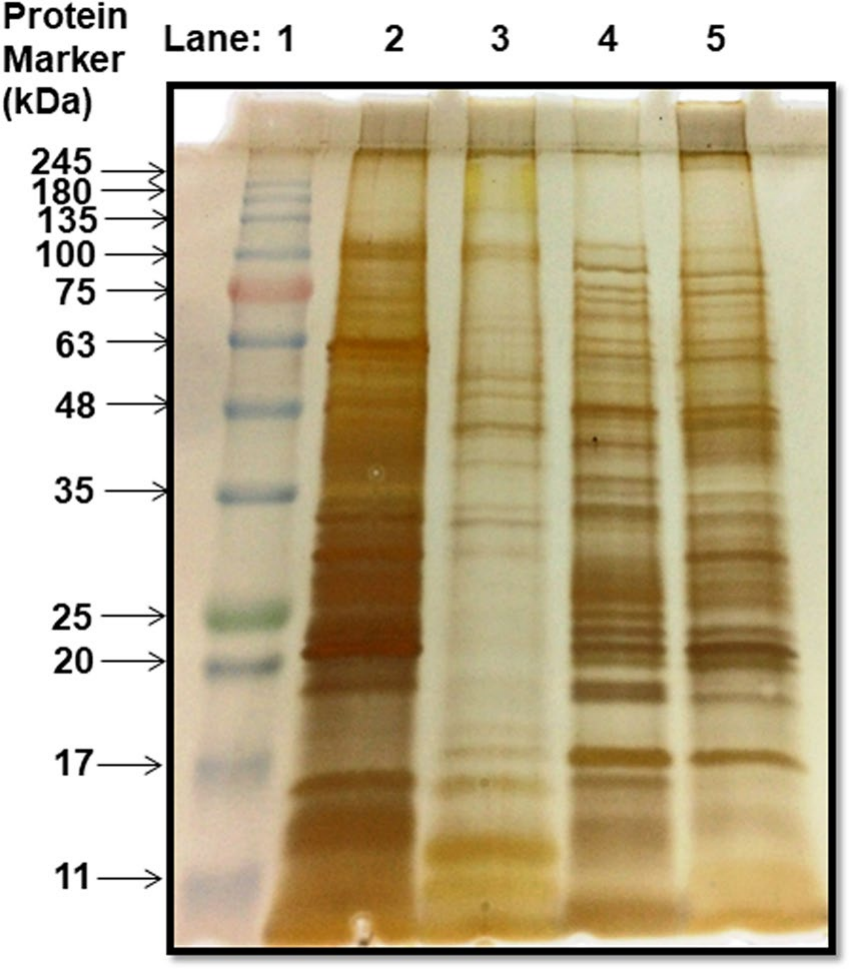
12% SDS-PAGE profile of purified protein extract from four types of human teeth. L1: Prestained molecular marker; L2: Molar protein extract; L3: Premolar protein extract; L4: Canine protein extract; L5: Incisor protein extract.

**Figure 2:**
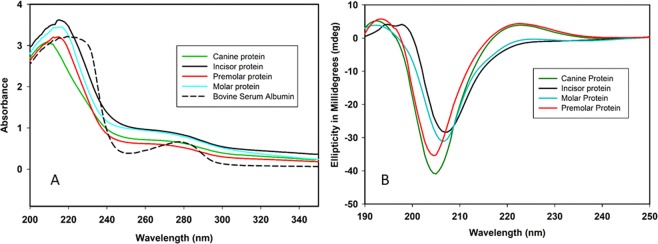
(**A**) Absorption spectra of all four protein extracts from human teeth along with BSA as a control protein. (**B**) Far-UV CD spectra of all four protein extracts from human teeth.

**Figure 3:**
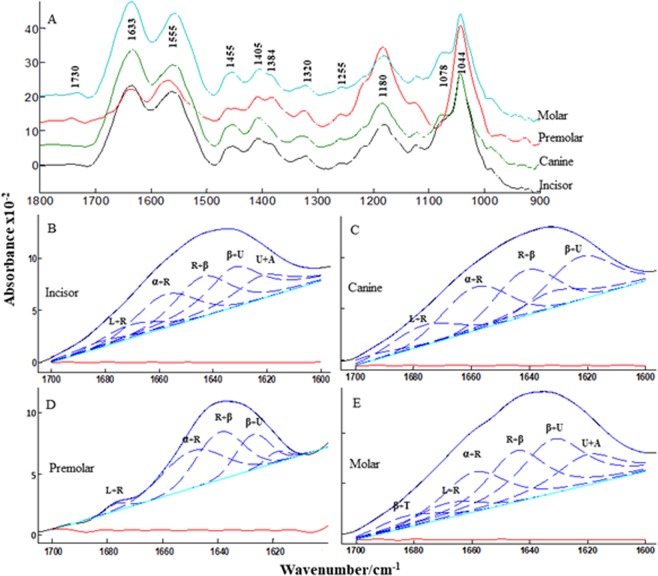
(**A**) Comparative infrared absorption spectra of all four protein extracts. All the spectra were baseline corrected and normalized. (**B**–**E**) Curve fitting of the amide I band of incisor (**B**) canine (**C**) premolar (**D**) and molar (**E**) protein extracts. Protein secondary structure content should read as α: alpha helix, β: beta sheets, L: loops, T: turns, R: Random, β’: Extended B sheets, U: Unstructured, A: Amino acid side chains. Each spectrum was baseline corrected and normalized to the equal band amplitudes.

Each type of protein extract was successfully purified upon demineralization of respective human teeth powder. These extracts were run on a 12% SDS–PAGE gel and subsequently stained using silver staining (Fig. 1). The gel showed clear bands of all four teeth protein extracts. As per our knowledge, this is the first report showing a differential proteome in four types of human teeth extracts i.e. Molar, Premolar, Incisor, and Canine. In terms of proteins, there is a clear qualitative as well as quantitative difference in all four of these, this may account for the differences present in their teeth shape which in turn, dictates functionality. Proteins such as bone morphogenic protein (BMP) is reported to control the shape of the crystal formed during teeth biomineralization^26^. These protein extracts constitute proteins from enamel, dentin, and cementum of individual type of teeth’s and some of these constituent proteins are known to be unstructured/disordered and can be characterized as IDP’s^12^. This view is well supported by Fig. 2A, which shows the ultraviolet spectral scanning of the different protein extracts and compares it with bovine serum albumin (BSA). This comparison signifies two things. Firstly, the proteins present in different extracts are devoid in aromatic residues like tyrosine, tryptophan, and phenylalanine, which itself is a typical trait of IDP’s and the primary reason for their “open” structures^27^. Secondly, the post-translational modifications like glycosylation of proteins in these extracts (also validated through FT-IR in Fig. 3A). All protein extracts showed higher absorbance above 300 nm as compared to BSA protein. In most cases, a protein remains optically inactive beyond 300 nm unless its interacting partner is a sugar molecule^28^ or the protein contains a higher number of charged residues^29^. In general, both are typical characteristics of IDP’s. Absorption around 220 nm is common in both which is a resultant of n-π* transitions of the peptide bond^30^.

### Circular dichroism (CD) of protein extracts from human teeth

CD spectroscopy has been used to infer the secondary structure content of proteins^31^. Far UV CD spectral absorbance of different protein extracts are shown in Fig. 2B. The negative ellipticity near 205 nm clearly suggests that the majority of the protein molecules are random coil /unstructured in nature^32,33^. In addition to this, some residual secondary structure in the form of polyproline II (PPII)-type structure is also seen. It is well known now that globally unfolded proteins may possess some regions where residual secondary structures reside^34^. This phenomenon is seen predominantly in the case of the premolar and canine protein extracts. The slightly positive band near 220 nm confirms the presence of these PPII type structures in the extracts^35^. However, in the case of molar and incisor protein extract, these bands at 220 nm (n-π* transitions) are absent, which shows that in the extracts PPII helix is not the dominant conformation. Although, collagen I itself is known to have a similar PPII helix structure due to the presence of high proline content. Several other tooth proteins, including amelogenin (which constitutes 95% of developing enamel matrix) has shown the presence of residual local secondary structures in the form of PPII type helices^36^.

Moreover, the data obtained from circular dichroism is in full agreement with the secondary structure calculation obtained by curve fitting of the amide I region (Fig. 3B–E).

### Fourier Transform Infrared Spectroscopy (FTIR) to characterize the human teeth

FTIR is a well-established technique for studying secondary structural characteristics of proteins and peptides^37^. Figure 3A shows the comparative FTIR profile of all four-different tooth protein extracts within the mid-infrared spectral region. The absorption range 1700–1600 cm^−1^ is attributed to the amide I vibration and arises mainly from the C=O stretching vibration with the minor contributions from protein backbone. The amide II mode range from 1600–1500 cm^−1^ is due to the out-of-phase combination of the NH in-plane bend and the CN stretching vibration. Amide III vibrations were also observed at ~1320 cm^−1^. In general, amide I vibration is most commonly used for calculating the secondary structural details as this region is less affected by the amino acid side chain^38^. Apart from amide group vibrations, these protein extracts also confirmed the presence of post-translational modification like glycosylation. Carbohydrate moiety whether monomeric or polymeric evoke strong band in 1200–900 cm^−1^ region. The bands observed at 1180 cm^1^ and 1044 cm^−1^ can be ascribed for C-O and C-C stretching vibration of carbohydrates respectively. Asymmetric and symmetric phosphate (P-O) vibrations are present at 1255 cm^−1^ and 1080 cm^−1^.

We also estimated the secondary structural content of these different protein extracts by performing curve fitting in the amide I region using the programs Kinetics^39^ and OPUS. The curve fitting is based on the second derivatives of the respective original absorption spectrum of all four extracts. This is a resolution enhancement method to separate the overlap bands. In the curve fitting, we input the wavenumber range for each peak based on the second derivative spectrum and then did the auto run by using the mixture of Lorentzian and Gaussian line shapes. This procedure resulted in the multi-component bands and their resultant was well overlapped (blue) with the original spectrum (black). The red line indicates the quality of the fits which is the subtracted resultant of the original and the overlapped band, we have observed straight lines in all the four extracts which implies a good fit. Figure 3B–E shows the fitted amide I component bands of all four protein extracts. We observed four prominent bands in these four protein extracts and their band assignments are as follows: band near 1678 cm^−1^ assigned to irregular structure (random coils)^40^, band near 1660 cm^−1^ assigned to alpha helices and irregular structure^10^, band near 1642 cm^−1^ assigned to irregular structures and beta sheets^38^, band near 1628 cm^−1^ assigned to irregular structure/PPII helix or extended beta pleated sheets^38^. We also assigned the band near 1690 cm^−1^ to beta turn and near 1615 cm^−1^ to irregular or side chains contribution. The secondary structure contents are summarized in Table 1. It can be clearly concluded that more than 50% of proteins constituting these extracts do not have stable secondary structure characteristics and the remaining are either in extended β structure or in turns and loops. A very small portion of the plot is indicating the presence of typically ordered structures like α-helix and β-sheets. The curve fitting results of amide I region of all four protein

**Table 1:** Estimation of the protein secondary structure contents of all the four teeth protein extracts.

Protein	Irregular structure	β-extended + irregular	Turns	α- helix + irregular
Incisor protein	24.5	49.0	6.0	20.5
Canine protein	23.0	39.0	10.0	28.0
Premolar protein	24.5	33.5	3.5	38.5
Molar protein	24.5	50.5	5.5	19.5

extracts underline primarily unstructured/random conformations, but still, some differences at the level of secondary structure contents can be observed. The absorbance around 1690 cm^−1^ is only present in the molar protein extract.

This probably means that β-turns frequency is higher in proteins present in the molar extracts as compared to others. Similarly, amino acid side chain absorption is visualized in molar and incisor protein extracts. However, the functional significance of these differences at the level of teeth is not possible.

### Bioinformatic predictions for structural disorder

Experimental approaches including CD and FTIR can only provide the gross structural estimates whether the protein is “mostly ordered” or “mostly disordered”, without shedding light on the residue-level resolution. The CH-plot (Supplementary Fig. 2) clearly reveals the residual level details of the disorderedness. Out of twenty investigated proteins, most lie on the left side of the borderline, which corresponds to a region of low mean hydrophobicity in addition to the presence of high values of mean net charge. These are the typical traits of disordered proteins. Moreover, we plotted the disordered pattern of some selected proteins involved in tooth biomineralization (Supplementary Fig. 3) which again demonstrate the perfect alliance among the two disorder predictors in almost all cases. It is now revealed through structure predictions, almost all proteins in the Swiss Protein database that have a role in mineralization are IDPs^13^.

### *In vitro* mineralization experiments in the presence of whole teeth protein extract

**Figure 4:**
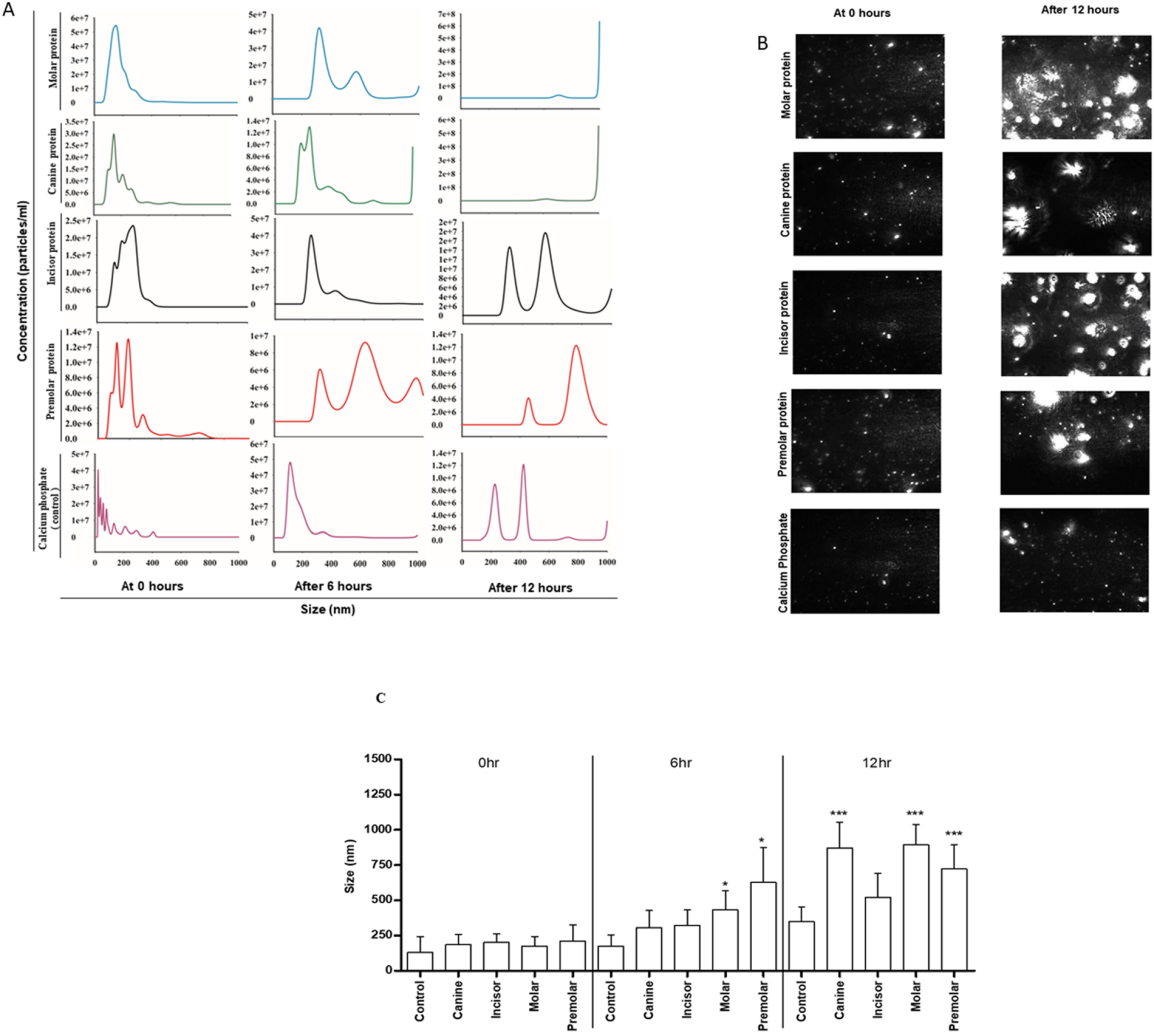
Nanoparticle tracking analysis (NTA) size measurement experiments to observe the biomineralization. (**A**) Size differences observed in molar, canine, incisor and premolar protein extract at 0 hours, 6 hours and 12 hours in the presence of calcium phosphate; control experiments with only calcium phosphate (absence of protein) were shown. (**B**) Representative image taken by NTA. All four teeth extracts at 0 hours and after 12 hours were shown. Control images of only calcium phosphate were also shown. (**C**) Statistical analysis of NTA experiments.

*In vitro* mineralization studies were done with the physiological stochiometric Ca/P molar ratio of 1.66 and in the presence of different protein extracts. Nanoparticle tracking analysis showed the size measurements at different time points, i.e. at 0 hours, and then after 6 and 12 hours of incubation of these mixtures at 37 °C. As evident from size measurement analysis, these protein extracts are elevating the crystallization process with increasing time. Figure 4A shows the results with all these four protein extracts from different teeth. We can clearly conclude that each of these protein extracts is involved in the promotion of hydroxyapatite crystallization. The control reaction with only calcium and phosphate is also shown. There is a steady increase in size which is a resultant of the forming calcium phosphate mineral phases, but the rate is much slower compared to the case with protein counterparts. This shows that the protein extracts promote the nucleation process and further increase the mineralization rates. In order to validate the specificity of our protein extracts, we used bovine serum albumin (BSA) protein (alone and in presence of calcium phosphate) as a random non-biomineralizing control protein (See Supplementary Fig. 4A) at different time points. We have also analyzed the protein aging effect (only protein extract and no calcium phosphate, Supplementary Fig. 4C). Finally, to remove collagen biased mineralization effects, a cisplatin induced nephrotoxic rat kidney tissue with elevated collagen content^41–43^ (confirmed by rat collagen I ELISA kit, see Supplementary Fig. 6) was also analyzed by mineralization experiments followed by NTA size measurements at different time points (Supplementary Fig. 4B).We found that none of these control experiments showed an increase in size with increased incubation time. Thus, proving the significance of IDPs in modulating the calcium phosphate mineralization. IDPs and their role in biomineralization has been recently reviewed by Adele Boskey’s group. Figure 4B shows the actual images for all these protein extracts as well as control at the molecular level from Nanosight instrument. Figure 4C shows the statistical significance of the data.

These differences in the shape and sizes of the hydroxyapatite crystals were also validated by polarization microscopy (Data not shown). Several other reports have shown the role of amelogenin in crystal nucleation, growth and regulation of shape and size of enamel mineralization^44,45^. In fact, Janet Oldak’s group has also looked into the cooperative effects enamel and amelogenin^46,47^ and amelogenin and ameloblastin^48^ on enamel mineralization and crystal formation^49^.

In order to emphasize the role of IDPs in teeth biomineralization, we successfully enriched our protein extract by virtue of their failure to be precipitated by 3% Perchloric acid treatment^25^ (Supplementary Fig. 5A). Moreover, this treated extract behaved similarly to the original, initial extract did in the *in vitro* mineralization followed by NTA size measurement experiments (Supplementary Fig. 5B). According to this, we can now conclude that the extracts do contain IDPs in the majority, and that IDPs do have prime roles in facilitating teeth biomineralization.

## Conclusion

In this study, we demonstrated the efficient isolation of proteins from different types of human teeth. Protein isolation from hard tissues like teeth, shell, and bone is generally a daunting task because of the need for removal of excessive inorganic ions and due to the low concentration of organic material. These protein extracts were used for structural studies which were carried out using FTIR and CD. Our studies show that most proteins exist in the unstructured state. The presence of such a flexible structure is inevitable in the case of tooth proteins. These structures provide the necessary flexibility which may be required for stabilization the ions or ion clusters, the critical nucleus, provide epitaxial sites for initial mineral deposition, and stabilize the crystal. As our protein extracts contain a heterogeneous mixture of proteins, our result shows the average structural data. Our spectral data are reproducible, and they overall represent the similar structural homogeneity among the teeth proteins. There is no single or group of proteins present in the mixture so as to indicate a different structural propensity. This is the first time that the structural homogeneity of proteins in different teeth is established.

**Figure 5:**
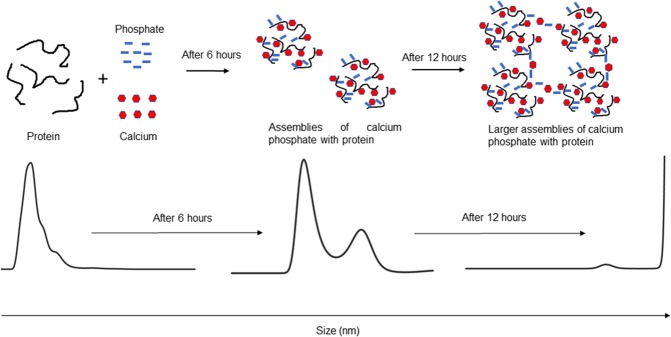
Schematic representation of *in vitro* mineralization and size measurement experiment.

We have also highlighted the importance of the residual level assessment of disorders through bioinformatics approaches like CH plot and disorder prediction software. Bioinformatics analysis was well correlated with the experimental data obtained from FTIR and CD. All of these marked the presence of unstructured proteins in protein extracts obtained from different types of human teeth. *In vitro* mineralization assays of hydroxyapatite in the presence of protein extracts showed that these protein extracts elevated the nucleation and foster the overall process of tooth biomineralization (Fig. 5).

Prior studies in this area have only investigated the role of individual proteins from the human tooth, addressing their structural disorder and contribution in biomineralization. Our study has focused on the whole extract from different types of human teeth so as to emulate the *in vivo* condition of tooth biomineralization.

## Supplementary information

Supplementary Information

## References

[1] Reith F (2009). Mechanisms of gold biomineralization in the bacterium Cupriavidus metallidurans. Proc Natl Acad Sci USA.

[2] Sumper M, Brunner E, Lehmann G (2005). Biomineralization in diatoms: characterization of novel polyamines associated with silica. FEBS Lett.

[3] DeVol RT (2015). Nanoscale Transforming Mineral Phases in Fresh Nacre. J Am Chem Soc.

[4] Paris O (2000). Analysis of the hierarchical structure of biological tissues by scanning X-ray scattering using a micro-beam. Cell Mol Biol (Noisy-le-grand).

[5] Jagr M (2014). Proteomics of human teeth and saliva. Physiol Res.

[6] Wright PE, Dyson HJ (1999). Intrinsically unstructured proteins: re-assessing the protein structure-function paradigm. J Mol Biol.

[7] Dunker AK, Silman I, Uversky VN, Sussman JL (2008). Function and structure of inherently disordered proteins. Curr Opin Struct Biol.

[8] Tompa P (2002). Intrinsically unstructured proteins. Trends Biochem Sci.

[9] van der Lee R (2014). Classification of intrinsically disordered regions and proteins. Chem Rev.

[10] Kumar S (2014). Infrared difference spectroscopy as a physical tool to study biomolecules. Applied Spectroscopy Reviews.

[11] Kelly SM, Jess TJ, Price NC (2005). How to study proteins by circular dichroism. Biochim Biophys Acta.

[12] Habchi J, Tompa P, Longhi S, Uversky VN (2014). Introducing protein intrinsic disorder. Chem Rev.

[13] Kalmar L, Homola D, Varga G, Tompa P (2012). Structural disorder in proteins brings order to crystal growth in biomineralization. Bone.

[14] Lee SL, Veis A, Glonek T (1977). Dentin phosphoprotein: an extracellular calcium-binding protein. Biochemistry.

[15] Prasad M, Butler WT, Qin C (2010). Dentin sialophosphoprotein in biomineralization. Connect Tissue Res.

[16] Wiedemann-Bidlack FB (2011). Effects of phosphorylation on the self-assembly of native full-length porcine amelogenin and its regulation of calcium phosphate formation *in vitro*. J Struct Biol.

[17] He G (2005). Spatially and temporally controlled biomineralization is facilitated by interaction between self-assembled dentin matrix protein 1 and calcium phosphate nuclei in solution. Biochemistry.

[18] Kwak SY (2014). Regulation of calcium phosphate formation by native amelogenins *in vitro*. Connect Tissue Res.

[19] Iijima M, Fan D, Bromley KM, Sun Z, Moradian-Oldak J (2010). Tooth enamel proteins enamelin and amelogenin cooperate to regulate the growth morphology of octacalcium phosphate crystals. Cryst Growth Des.

[20] Goormaghtigh E, Ruysschaert JM, Raussens V (2006). Evaluation of the information content in infrared spectra for protein secondary structure determination. Biophys J.

[21] Valdivia AA, Barth A, Batista YR, Kumar S (2013). Characterization of recombinant antibodies for cancer therapy by infrared spectroscopy. Biologicals.

[22] Kyte J, Doolittle RF (1982). A simple method for displaying the hydropathic character of a protein. J Mol Biol.

[23] Uversky VN (2002). Natively unfolded proteins: a point where biology waits for physics. Protein Sci.

[24] Dosztanyi Z, Csizmok V, Tompa P, Simon I (2005). IUPred: web server for the prediction of intrinsically unstructured regions of proteins based on estimated energy content. Bioinformatics.

[25] Cortese MS, Baird JP, Uversky VN, Dunker AK (2005). Uncovering the unfoldome: enriching cell extracts for unstructured proteins by acid treatment. J Proteome Res.

[26] Jernvall J, Thesleff I (2012). Tooth shape formation and tooth renewal: evolving with the same signals. Development.

[27] Yizhi Z, Helene L, Antoine S, Regine L, Brigitte G (2018). Exploring intrinsically disordered proteins in Chlamydomonas reinhardtii. Sci Rep.

[28] Berrens LABE (1966). The influence of sugars on the UV absorption spectrum of proteins. Recueil des Travaux Chimiques des Pays‐Bas.

[29] Prasad S (2017). Near UV-Visible electronic absorption originating from charged amino acids in a monomeric protein. Chem Sci.

[30] C R. Cantor and Schimmel, P. R. *Biophysical chemistry: Part II ‘Techniques for the study of biological structure and function*. (Freeman, W. H. 1981).

[31] Greenfield NJ (2006). Using circular dichroism spectra to estimate protein secondary structure. Nat Protoc.

[32] Receveur-Brechot V, Bourhis JM, Uversky VN, Canard B, Longhi S (2006). Assessing protein disorder and induced folding. Proteins.

[33] Delak K (2009). The tooth enamel protein, porcine amelogenin, is an intrinsically disordered protein with an extended molecular configuration in the monomeric form. Biochemistry.

[34] Ishijima J, Nagasaki N, Maeshima M, Miyano M (2007). RVCaB, a calcium-binding protein in radish vacuoles, is predominantly an unstructured protein with a polyproline type II helix. J Biochem.

[35] Lopes JL, Miles AJ, Whitmore L, Wallace BA (2014). Distinct circular dichroism spectroscopic signatures of polyproline II and unordered secondary structures: applications in secondary structure analyses. Protein Sci.

[36] Lakshminarayanan R, Fan D, Du C, Moradian-Oldak J (2007). The role of secondary structure in the entropically driven amelogenin self-assembly. Biophys J.

[37] Krimm S, Bandekar J (1986). Vibrational spectroscopy and conformation of peptides, polypeptides, and proteins. Adv Protein Chem.

[38] Barth A (2007). Infrared spectroscopy of proteins. Biochim Biophys Acta.

[39] Baldassarre M, Scire A, Fiume I, Tanfani F (2011). Insights into the structural properties of D-serine dehydratase from Saccharomyces cerevisiae: an FT-IR spectroscopic and in silico approach. Biochimie.

[40] Goormaghtigh E, Cabiaux V, Ruysschaert JM (1994). Determination of soluble and membrane protein structure by Fourier transform infrared spectroscopy. I. Assignments and model compounds. Subcell Biochem.

[41] Sahu AK (2019). Mangiferin attenuates cisplatin-induced acute kidney injury in rats mediating modulation of MAPK pathway. Mol Cell Biochem.

[42] Li, H. Y., Yang, S., Li, J. C. & Feng, J. X. Galectin 3 inhibition attenuates renal injury progression in cisplatin-induced nephrotoxicity. *Biosci Rep* **38**, 10.1042/BSR20181803 (2018). 10.1042/BSR20181803 PMC6435560 30455396

[43] Taguchi T, Nazneen A, Abid MR, Razzaque MS (2005). Cisplatin-associated nephrotoxicity and pathological events. Contrib Nephrol.

[44] Fincham AG, Moradian-Oldak J, Simmer JP (1999). The structural biology of the developing dental enamel matrix. J Struct Biol.

[45] Wang L, Guan X, Du C, Moradian-Oldak J, Nancollas GH (2007). Amelogenin Promotes the Formation of Elongated Apatite Microstructures in a Controlled Crystallization System. J Phys Chem C Nanomater Interfaces.

[46] Fan D (2011). The cooperation of enamelin and amelogenin in controlling octacalcium phosphate crystal morphology. Cells Tissues Organs.

[47] Bouropoulos N, Moradian-Oldak J (2004). Induction of apatite by the cooperative effect of amelogenin and the 32-kDa enamelin. J Dent Res.

[48] Mazumder P, Prajapati S, Lokappa SB, Gallon V, Moradian-Oldak J (2014). Analysis of co-assembly and co-localization of ameloblastin and amelogenin. Front Physiol.

[49] Boskey AL, Villarreal-Ramirez E (2016). Intrinsically disordered proteins and biomineralization. Matrix Biol.

