# Publisher Correction: Characterization of protein extracts from different types of human teeth and insight in biomineralization

**DOI:** 10.1038/s41598-019-53780-4

**Published:** 2019-11-21

**Authors:** Vaibhav Sharma, Alagiri Srinivasan, Ajoy Roychoudhury, Komal Rani, Mitali Tyagi, Kapil Dev, Fredrik Nikolajeff, Saroj Kumar

**Affiliations:** 1 Department of Biophysics, All India Institute of Medical Sciences (AII MS), New Delhi, India; 2 Department of Biochemistry, Jamia Hamdard University, New Delhi, India; 3 Department of Oral and Maxillofacial Surgery, Center for Dental Education and Research (CDER), All India Institute of Medical Sciences (AII MS), New Delhi, India; 4 Department of Engineering Science, Uppsala University, Uppsala, 75105 Sweden; 5 Department of Biotechnology, Jamia Milia Islamia, New Delhi, India

Correction to: *Scientific Reports* 10.1038/s41598-019-44268-2, published online 27 June 2019

In the original version of this Article, Kapil Dev was incorrectly listed as the corresponding author. The correct corresponding author for this Article is Saroj Kumar. Correspondence and request for materials should be addressed to sarojgupta.k@gmail.com

Additionally, in the Supplementary Information file originally published with this Article, Kapil Dev was omitted from the author list.

These errors have now been corrected in the HTML and PDF versions of the Article and in the accompanying Supplementary Information file.

