# Supplementary material for: Characterization of protein extracts from different types of human teeth and insight in biomineralization
Source: Sci Rep. 2019 Jun 27;9:9314. doi: 10.1038/s41598-019-44268-2 (PMC6597790; doi:10.1038/s41598-019-44268-2)
Supplement: Supplementary file 1 — Supplementary Information [file 41598_2019_44268_MOESM1_ESM.docx]

**Supplementary Information**

**Characterization of protein extracts from different types of human teeth and insight in biomineralization**

Vaibhav Sharma^1^, Alagiri Srinivasan^2^, Ajoy Roychoudhury^3^ Komal Rani^1^, Mitali Tyagi^1^, Kapil Dev^5^, Fredrik Nikolajeff ^4^, Saroj Kumar^1*^

^1^ Department of Biophysics, All India Institute of Medical Sciences(AIIMS), New Delhi, India.

^2^ Department of Biochemistry, Jamia Hamdard University, New Delhi, India.

^3^ Department of Oral and Maxillofacial Surgery, Center for Dental Education and Research (CDER), All India Institute of Medical Sciences(AIIMS), New Delhi, India.

^4^Department of Engineering Science, Uppsala University, Uppsala 75105 (Sweden)

^5^ Department of Biotechnology, Jamia Milia Islamia, New Delhi, India

*Corresponding author: [sarojgupta.k@gmail.com](mailto:sarojgupta.k@gmail.com); Tel: +91-011-26594240


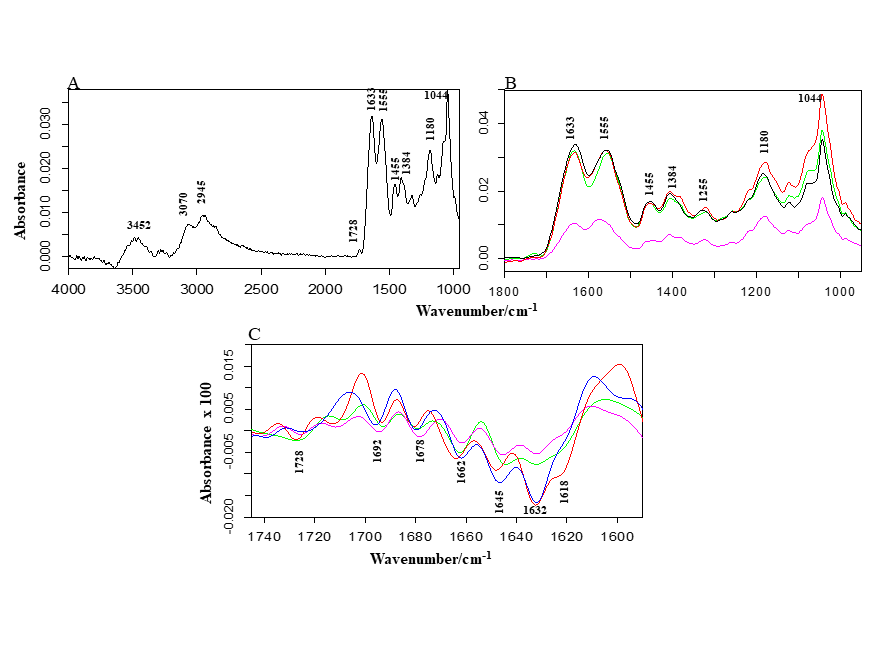


Supplementary Figure:1

**
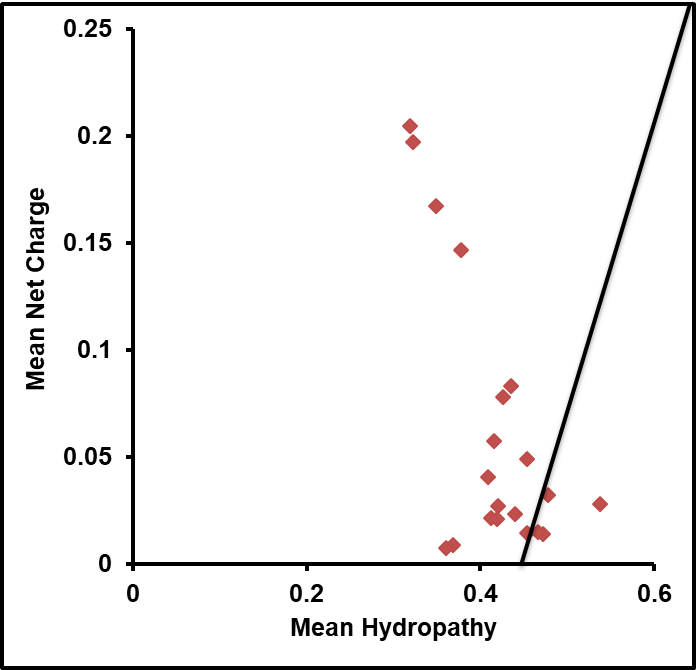
**

Supplementary Figure:2

**
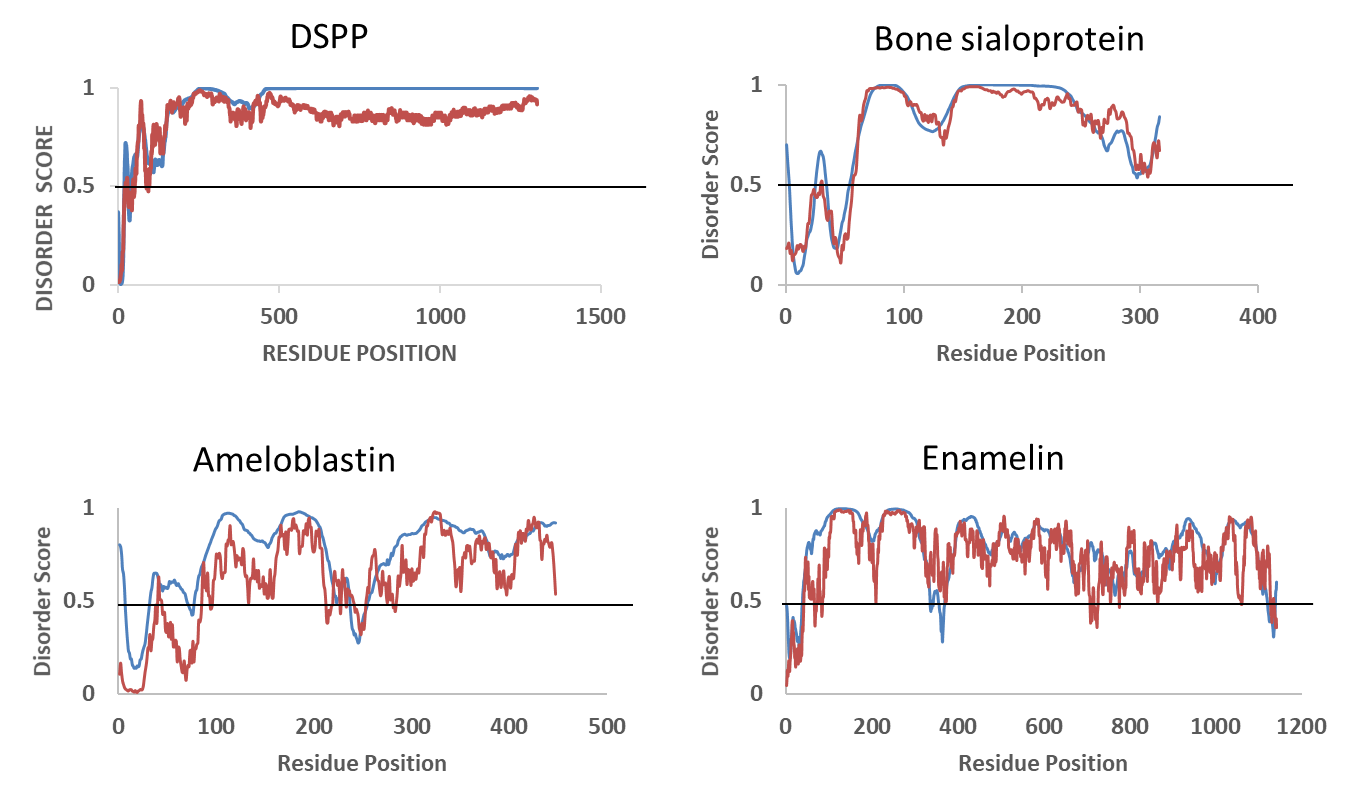
**

**
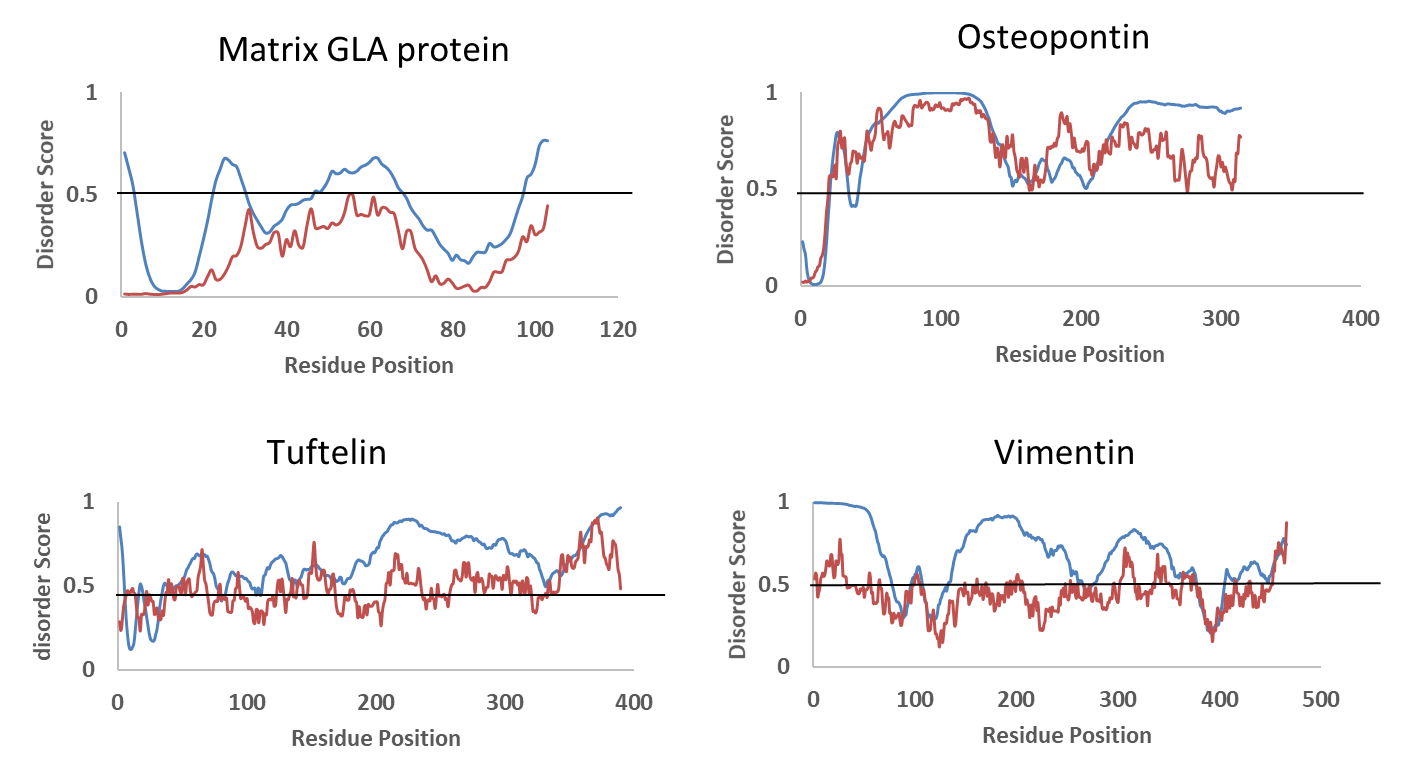
**

**
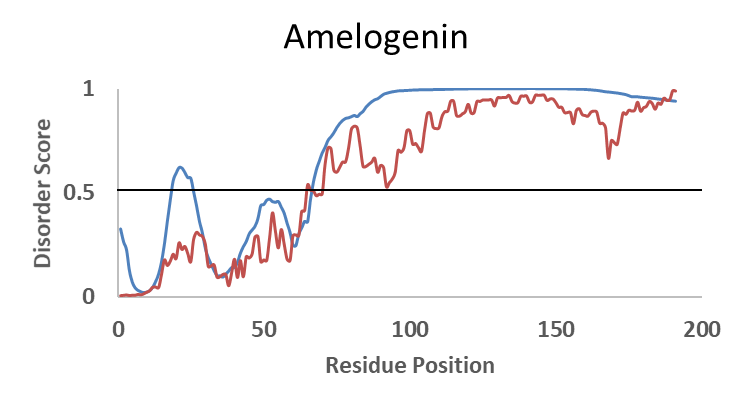

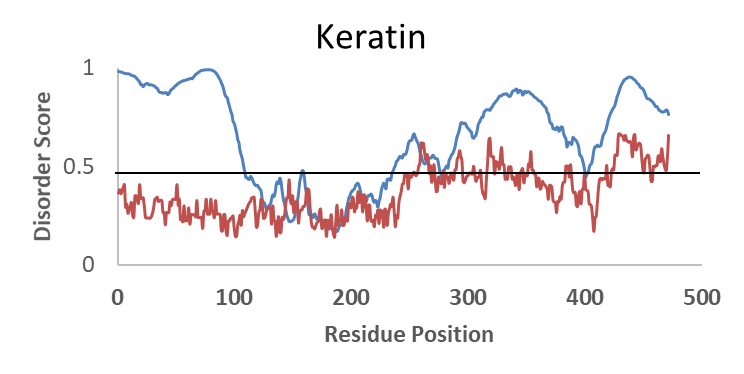
**

**
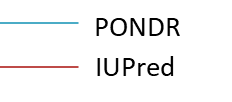
**

Supplementary Figure:3

**
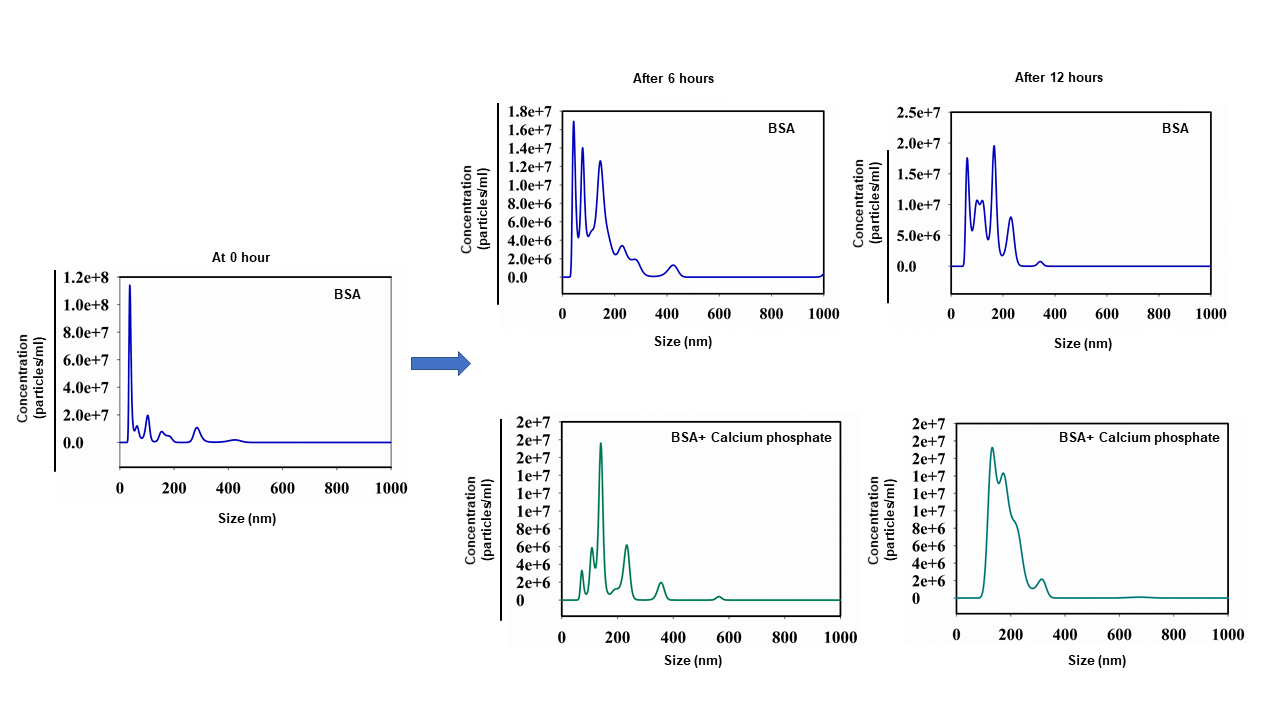
**

**B**

**A**

**
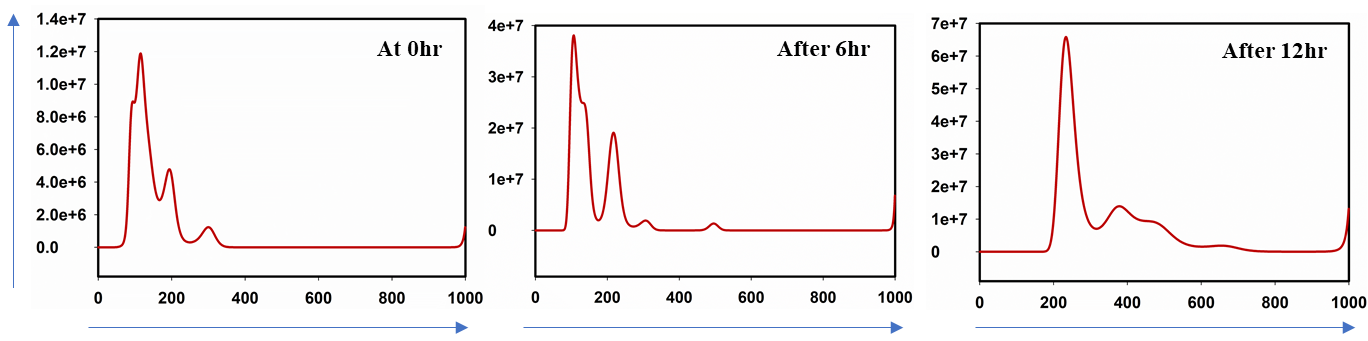
**

**Concentration (particles/ml)**

**Size (nm)**

**C**

**
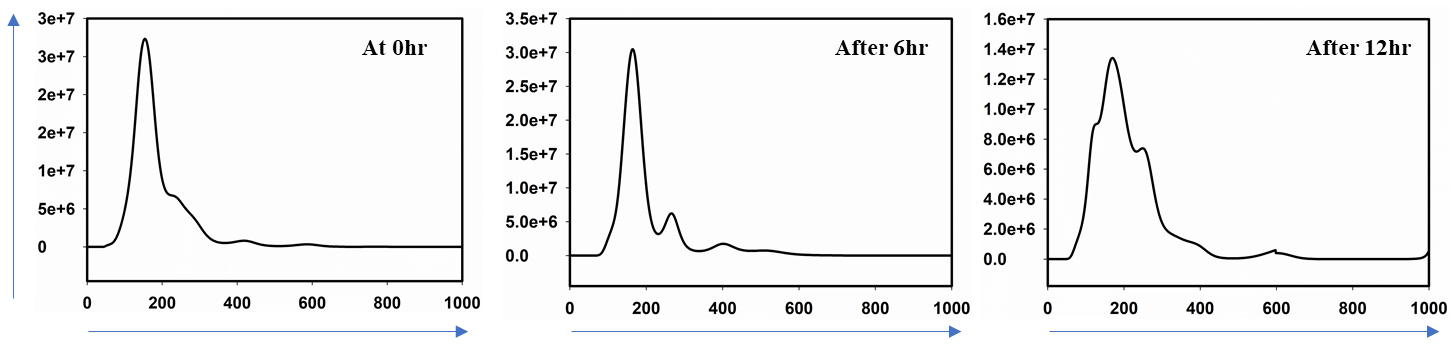
**

**Concentration (particles/ml)**

**Size (nm)**

Supplementary Figure:4

**A**

**
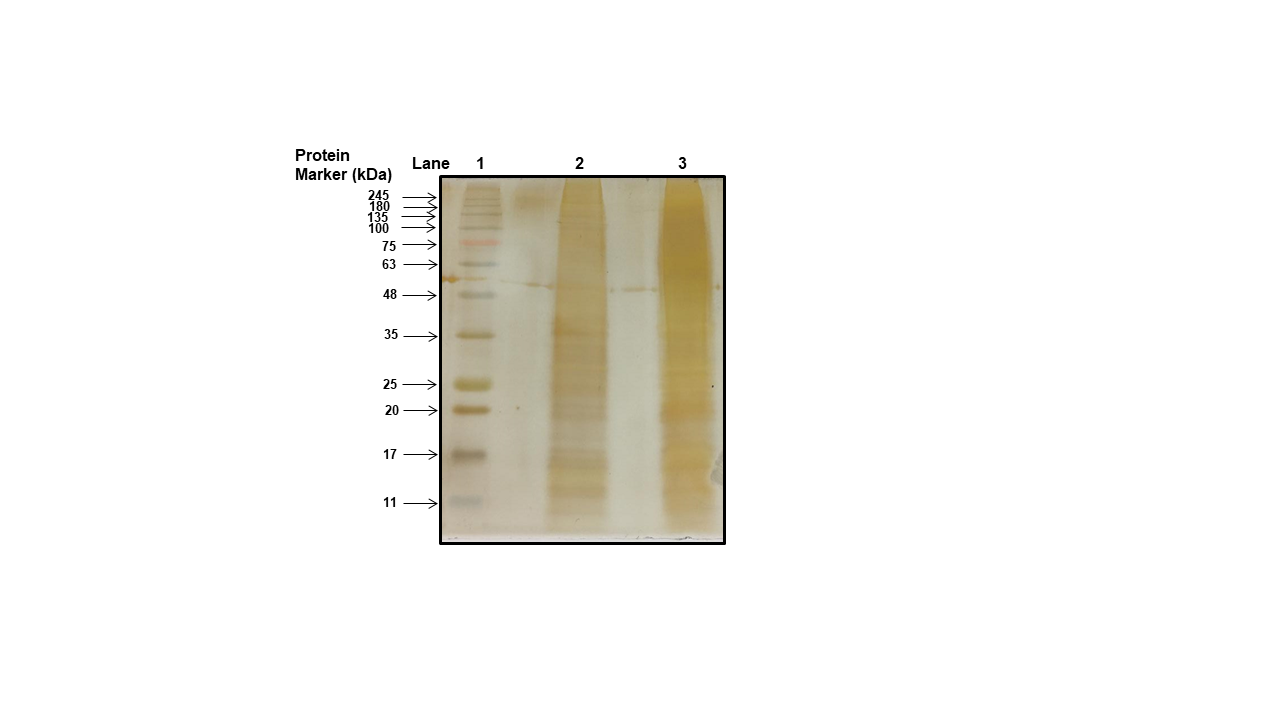
**

**B**


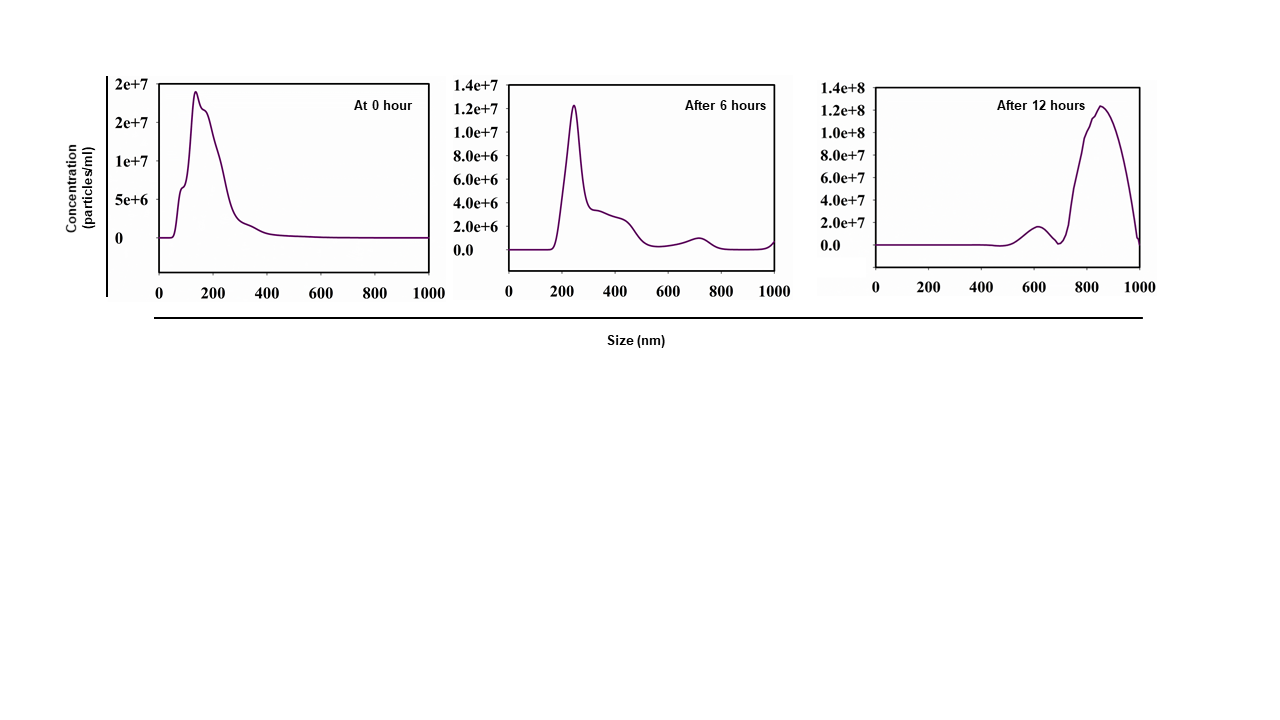


**C**

**
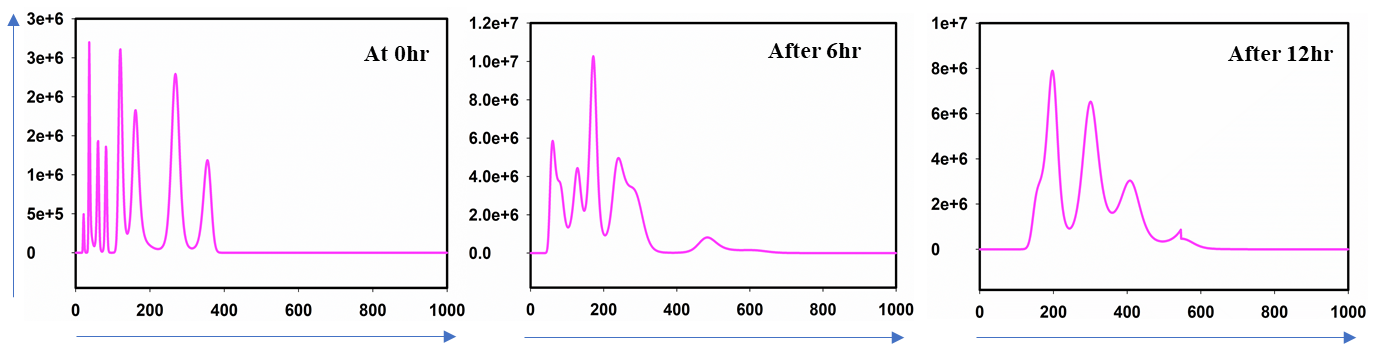
**

**Concentration (particles/ml)**

**Size (nm)**

Supplementary Figure:5

Supplementary Figure:5

**
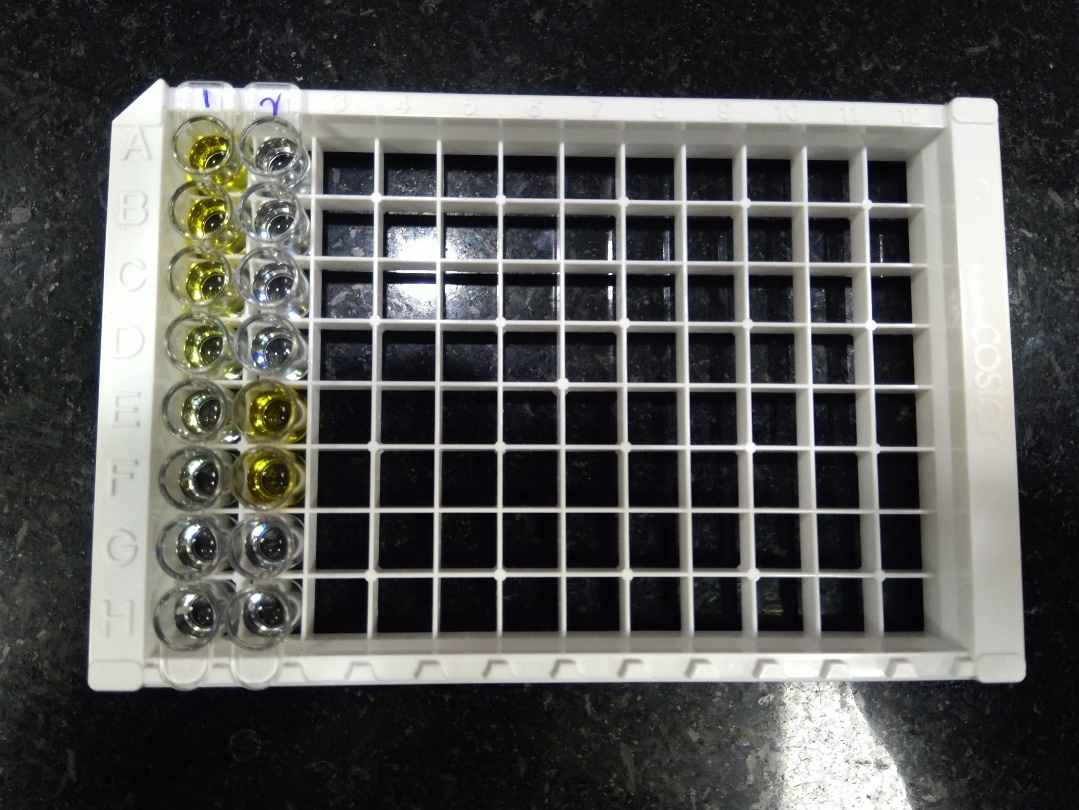
**

Supplementary Figure:6

**Supplementary Figure:1** A) Infrared absorbance spectrum of a tooth protein in mid-infrared region (4000 to 900 cm^-1^)

B) Raw data of infrared absorbance spectra of all four teeth protein extracts.

C) Second derivative of the infrared absorbance spectra of all four teeth protein extracts presented in B .

**Supplementary Figure: 2** Charge hydropathy (CH) plot between mean hydropathy and mean net charge. The solid line represents the border between intrinsically disordered and native proteins.

**Supplementary Figure: 3** Disorder pattern of selected proteins from Human teeth. The proteins are predicted to be mostly or fully disordered by IUPred (red line) and PONDR© VSL2 (blue line) predictors. Residues above the 0.5 thresholds are considered disordered.

**Supplementary Figure:4** Control Nanoparticle tracking analysis (NTA) experiments

A) BSA (alone) and BSA+ Calcium phosphate.

B)Cisplatin injured rat kidney tissue (excessive collagen content)+Calcium phosphate

C) Molar protein ageing with time (Only protein control, No calcium phosphate)

**Supplementary Figure:5** A) 12% SDS Gel electrophoresis with silver stain. Lane:1 Prestained marker; Lane:2 Canine extract; Lane:3 Insoluble extract left after 3% perchloric (PCA) treatment of Canine extract.

B) Size measurement experiment of the insoluble left out extract after 3% PCA treatment (Canine extract) at 0 hour, 6 hours and 12 hours.

C) Size measurement experiment of the pelleted extract after 3% PCA treatment (Canine extract) at 0 hour, 6 hours and 12 hours.

Supplementary Figure: 6 Rat Collagen I ELISA Plate assay (CUSA BIO)

Column1: Standard - 5000pg/ml(A1) to 0pg/ml (H1)

Column:2 A1-D1- 4 different tooth protein extracts

E1,F1- Cisplatin injured Rat kidney isolate

G1,H1-Control
